# Barriers to Hospital Deliveries among Ethnic Minority Women with Religious Beliefs in China: A Descriptive Study Using Interviews and Survey Data

**DOI:** 10.3390/ijerph13080815

**Published:** 2016-08-11

**Authors:** Peige Song, Chuyun Kang, Evropi Theodoratou, Neneh Rowa-Dewar, Xuebei Liu, Lin An

**Affiliations:** 1 Department of Child, Adolescent and Women’s Health, School of Public Health, Peking University, Beijing 100191, China; peigesong@hsc.pku.edu.cn (P.S.); kangchymail@163.com (C.K.); liuxuebei87@126.com (X.L.); 2 Centre for Population Health Sciences, University of Edinburgh, Edinburgh EH8 9AG, UK; e.theodoratou@ed.ac.uk (E.T.); Neneh.Rowa-Dewar@ed.ac.uk (N.R.-D.)

**Keywords:** barriers, hospital delivery, poor ethnic minority, China

## Abstract

**Background:** China has made great progress in improving hospital delivery-the coverage of hospital delivery has increased to above 95% in most regions- some regions lag behind owing to geographic and economic inequality, particularly the poor ethnic minority areas of the Sichuan Province. This study explores factors which may influence hospital delivery from multiple perspectives, with implications for practice and policy. **Methods:** A framework analysis approach was used to identify and categorize the main barriers and levers to hospital delivery. Our analysis draws on basic information from the sampled counties (Butuo and Daofu). **Results:** The hospital delivery rate was below 50% in the two sampled areas. In both areas, the “New Rural Cooperative Medical Scheme” and “Rural hospital delivery subsidy” were introduced, but only Butuo county had a transportation subsidy policy. Socioeconomically disadvantaged women in both counties who delivered their babies in hospitals could also apply for financial assistance. A lack of transport was among the main reasons for low hospital delivery rates in these two counties. Furthermore, while the hospital delivery costs could be mostly covered by “New Rural Cooperative Medical Scheme” or “Rural Hospital Delivery Subsidy”, reimbursement was not guaranteed. People in Daofu county might be affected by their Buddhism religion for hospital delivery. Women in Butuo following the Animism religion would refuse delivery in hospitals because of language barriers. Traditional lay beliefs were the main factor that influenced hospital delivery; their understandings of reproductive health varied, and many believed that childbirth should not be watched by strangers and that a home delivery was safe. **Conclusions:** This study has highlighted a number of barriers and levers to hospital delivery in rural poor ethnic minority areas which could inform and improve the access and rate of hospital delivery rate; thereby reducing health inequalities in maternal and child health in China.

## 1. Introduction

Globally, approximately 800 women die each day from causes related to pregnancy and childbirth, 99% of which occur in developing countries, and most can be avoided with immediate and effective professional care during and after delivery [1,2]. Although home births could be safe, hospital delivery is advocated in most countries as obstetric emergencies can be managed more efficiently [3]. Consequently, hospital delivery is recognized as an effective strategy in reducing maternal and perinatal mortality, and improving the health and well-being of the mothers and newborns [4,5].

The Chinese government began to encourage hospital delivery from July 1995 [3], with the safe motherhood initiative in 40 of the poorest counties supported by the United Nations International Children's Emergency Fund (UNICEF) in 1999 [6]. Since 2000, China began to perform “Reducing maternal mortality and eliminating newborn tetanus” in the rural counties of 12 central and western provinces, gradually extended it to 22 provinces in 2011, covering around 830 million population [7]. In this program, the most important intervention was to increase of hospital delivery rate by waiving in-patient charges for hospital deliveries and improving the quality of local maternity services [8]. At the same time, home births were defined as “illegal” and traditional birth attendants’ licenses were suspended by local health administrative departments [9]. In addition, the “New Rural Cooperative Medical Scheme” (NRCMS), a new health insurance program targeting rural residents, was initiated in 2003. It operates at the county level and, as a match fund, comprises a central government subsidy, county government contributions, and individual contributions (poor rural residents’ individual contributions are waived) [10]. This scheme partially reimburses hospital delivery costs [3]. A further and targeted program named “Rural Hospital Delivery Subsidy” (RHDS) was initiated in 2009 to reimburse hospital delivery fees for rural women aiming to achieve more than a 95% hospital delivery rate [11,12]. RHDS provides 500 Yuan per rural woman delivering at hospital and should be combined with NRCMS to ease the financial burden of rural hospital delivery [13].

All of these initiatives resulted in an increase in overall hospital delivery rate in China from 72.9% in 2000 to 97.8% in 2010, above 95% in most regions. Maternal mortality decreased from 53.0 per 100,000 live births in 2000 to 30.0 per 100,000 live births in 2010 and neonatal mortality decreased from 22.8 per 1000 live births in 2000 to 8.3 per 1000 live births in 2010 [14].

**Figure 1:**
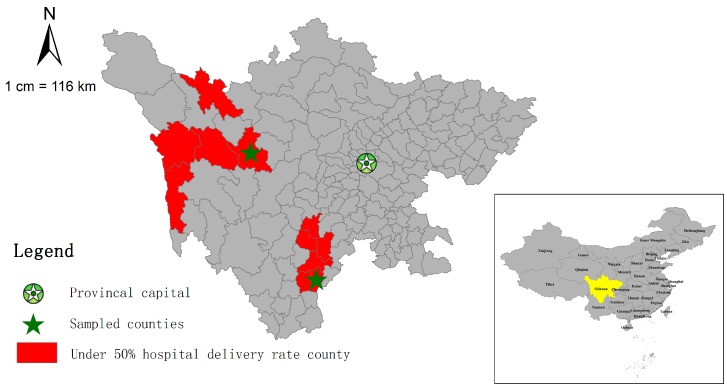
Location of the sampled counties.

Despite such nationwide progress, the national maternal and child health (MCH) statistics annual report system (not publicly accessible), identifies some regions are still lagging behind. In 2011, there were 188 counties (6.6% of all counties) with a hospital delivery rate of less than 90%, and among them, 30 (1.0% of all counties) had rates below 50%. All of these were in the Tibet Autonomous Region and Sichuan Province. Nineteen of the 73 counties (26.0%) in Tibet had a hospital delivery rate below 50%. Some studies show that inequity also exists in some subgroups of populations, such as low-income households, rural women, and ethnic minorities [15]. Eleven of the 181 counties in Sichuan (6.1%) had a hospital delivery rate below 50%, demonstrating the inequality gap in this province since the average rate for the whole Sichuan Province was as high as 92.5%. These 11 counties were located in the west rural ethnic minority areas of Sichuan Province (Figure 1). Sichuan Province is in Southwest China covering 485,000 square kilometers and occupying 5.1% of the total area of China. It is also where 55 of the all 56 ethnic minorities in China reside [16]. According to the sixth National Population Census in November 2010, the total population of Sichuan Province was 80,418,200, which made it the fourth most populous province in China [17].

Developing specific in-depth local strategies for the regions is of importance with low hospital delivery coverage [7]. There are numerous factors affecting the utilization of maternal care including maternal, family and social factors, policy interventions, and health facility factors [18,19,20]. While there are studies analyzed the demanding factors [3,21], understanding remains limited for social and provision factors.

This study aims to explore why various policy and practice interventions aimed at increasing hospital delivery rates have not been effective compared to other regions of Sichuan Province from policy, institutional, and social factors. It also aims to draw out the implications for policy and practice to reduce maternal and child health inequalities.

## 2. Materials and Methods

### 2.1. Settings

In the Sichuan Province, all of the 11 counties with hospital delivery rates lower than 50% are located within two ethnic minority autonomy prefectures, six are located in the Liangshan Yi ethnic minority autonomy prefecture (Animism religion area) and five in the Ganzi Tibetan ethnic minority autonomy prefecture (Buddhism religion area). To explore the different problems and needs between different ethnic minorities, we randomly selected one county with below-50% hospital delivery rate from each ethnic prefecture (Butuo and Daofu). Within Butuo and Daofu, all the towns were classified into respective-high and respective-low strata based on hospital delivery rates, and then one town was sampled from each stratum. Finally, two counties and four townships were sampled for this study. We used the original name—Butuo county and Daofu county (Figure 1)—to refer to the two counties, and used the code names (B1, B2) and (D1, D2) to represent the four townships in Butuo county and Daofu county, respectively.

### 2.2. Data Collection

This study was conducted in September 2012 using both quantitative and qualitative methods: questionnaires (two, one in each county), interviews (12) and focus group discussions (two; one in each county). The questionnaires gathered basic information of each county and each township from senior members (head or vice-heads) of the local health administrative sectors.

Twelve in-depth interviews lasting approximately 30–60 min each were conducted with: (1) local government health administrators familiar with the situation of hospital delivery (two in each county; four in total); (2) health facility administrators/medical staff (three in each county; six in total); and (3) village doctors (one in each county; two in total) to explore issues related to transportation and geography and health policy and practice.

Two focus group discussions lasting 1–2 h each were conducted in local township hospitals with seven and eight local village women, respectively, to explore economic, cultural, family (including family income and family relationships), and personal issues (including knowledge and workloads) until saturation. Participants were volunteers aged 20–40 years and were all within three years’ post-partum. The majority of the participants were Yi and Tibetan speakers, so questions were asked in the local language and responses were translated into Mandarin by educated translators. Notes were taken by researchers in Mandarin Chinese. Translators were anonymous and not from the local governments or health facilities. All the interviews and focus group discussions were audio-recorded and transcribed verbatim in Mandarin Chinese.

**Figure 2:**
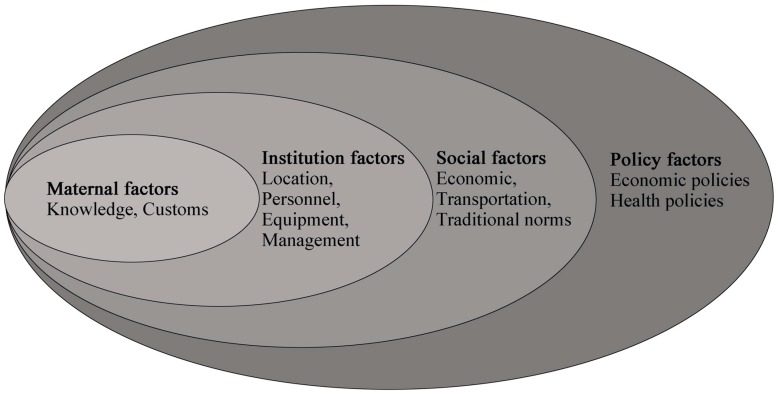
The framework of the utilization of hospital delivery care.

The qualitative data were analyzed using NVivo 8 text analysis software (QSR International, Melbourne, Australia) with initial coding based on the analysis framework (Figure 2) preset by a pilot expert meeting (including six experts from public health, health policy, and epidemiology disciplines until saturation) to understand the key barriers of hospital delivery in these counties.

### 2.3. Ethical Approval

The research was approved by the National Maternal and Child Health Annual Report office (in charge of all the MCH report departments nationwide), local health departments and heads of the appropriate health facilities. Participation was voluntary and informed, and provided verbal consent as most participants were illiterate people. Individual data were in an anonymous format for recording and analysis, no additional ethical approval was required.

## 3. Results

### 3.1. Characteristics of the Sampled Counties

#### 3.1.1. Butuo County

Butuo county is located in Liangshan Yi ethnic minority autonomy prefecture (Figure 1). Most of the areas in Butuo county are mountain areas, with an average altitude of 2300 m and agriculture is the main industry. As listed in Table 1, the total population of Butuo was 170,000 in 2011, and the population density was 102/km^2^. This county is one of the poorest counties in China, there are no roads in two of the 20 townships and in 63 of the 190 villages.

The total number of live births in Butuo was 3370 in 2011, and only 22.1% of these births (745 births) took place in a hospital. There were 37 hospitals, seven with delivery and maternal facilities, including the county people’s hospital, the county MCH center, and five township hospitals. Of these seven health facilities, in only three hospitals pregnant women were admitted in 2011, with no deliveries in the other four hospitals. Of the 745 hospital deliveries in 2011, 88% took place in the county people’s hospital, with the remaining 12% of hospital deliveries distributed in two township hospitals. Only three women gave birth in the county MCH center.

Traditional midwives assisted some home deliveries but there is no legally licensed birth attendant attending home deliveries in this county. Their patients were mainly their relatives or neighbors, and payment was low or non-existent. Some of these midwives graduated from nursing school and provided birth assistance for years before the national action of home delivery prohibition was introduced. Some still used the midwifery packages given by the local county health bureau several years ago, and asserted their practice as new-method delivery assistance. A large number of women who gave birth at home were assisted by older women in their families, such as mothers, mothers-in-law, and sisters, the majority of whom had no formal medical education or training and used non-sterilized midwifery packages.

#### 3.1.2. Daofu County

Daofu county is located in the Ganzi Tibetan ethnic minority autonomy prefecture (Figure 1). Most of the areas in Daofu county are mountainous, with the average altitude of 3500 m and agriculture and animal husbandry the main industries. As shown in Table 1, the total population was 55,000 in 2011, and the population density was 8/km^2^. Although Daofu county is not a national level poverty county like Butuo county, its per capita income was lower than that of the Butuo county (3303 vs. 3575 Yuan). There are 22 townships, all of which have roads, but four of the 158 villages do not have roads.

A total of 539 live births were recorded in the county in 2011, and 47.1% of babies (254 births) were delivered at hospital. Three out of 28 hospitals had midwives, including the county people’s hospital, the county MCH center and one township hospital. In 2011, there were no deliveries in the county MCH center. Ten deliveries took place in the township hospital and 244 in the county people’s hospital.

Similar to Butuo county, in Daofu county, pregnant women who gave birth at home were usually assisted by the elder women of the family or illegal midwives and most were not given modern delivery assistance tools.

### 3.2. Policy Factors for Hospital Delivery

#### 3.2.1. New Rural Cooperative Medical Scheme

In the sampled counties, the individual fee for NRCMS was 50 Yuan per year. Ninety-five percent of the rural population was enrolled in NRCMS in Butuo county, while in Daofu county, the coverage percentage was 97%. There were mainly two reasons for nonparticipation in NRCMS; either migrating to another province for work or reluctance to pay the NRCMS fee.

#### 3.2.2. Rural Hospital Delivery Subsidy

There was no difference between the two counties in RHDS policy implementation for rural women and the amount of subsidy was 500 Yuan per capita. Those granted it were resident women with rural household registration who delivered in hospital (regardless of the hospital location, the number of past childbirths, NRCMS participation or delivery way). Women were asked to apply for reimbursement at the county MCH center bringing along the birth certificate (acting as a hospital delivery proof). Women who gave birth at home were not granted an RHDS.

#### 3.2.3. Promoting Hospital Delivery in Sichuan Ethnic Minority Regions Program

This is a program launched by the Departments of Health and Finance, and by the Women’s Federation of Sichuan Province from May 2010. Since the introduction of this program, the MCH center of Butuo county received 1.5 million Yuan, and the people’s hospital of Daofu county also received a million Yuan for hospital construction. This program also provides transportation subsidy (TS) for hospital deliveries. In Butuo county, every rural woman giving birth in hospital receive 100 Yuan as a transportation allowance and if she was sent by a village doctor, the doctor would receive 50 Yuan as an escort reward. However, women were often reluctant to inform village doctors about their pregnancies in case of being persuaded to give birth in hospitals, so that even when village doctors were required and willing to escort few women actually went to hospital. In Daofu county, there was no TS for pregnant women or reward for an escort by the village doctor; consequently, the women’s families were responsible for the cost of transfer to hospital.

#### 3.2.4. Other Policies

In these two counties, NRCMS and RHDS were the two main policies responsible for covering the majority of hospital delivery expenditure. Socioeconomically disadvantaged women delivered in hospital could also apply for Poverty Financial Assistance (PFA) from the county civil affairs department. The amount of this subsidy is not fixed as it depends on the financial situation of the household.

### 3.3. Social Factors for Hospital Delivery

#### 3.3.1. Transportation

Based on the interviews, transportation was among the leading reasons for low hospital delivery rate in these two counties. As mentioned above, two townships and 63 villages in Butuo had no roads suitable for vehicles. Although all the townships in Daofu were suitable for vehicular transport, four out of 158 villages were not. Despite the road barrier, the lack of motor vehicles was a major obstacle for transfer to hospital. Many villages had no vehicles, so pregnant women would have to walk or travel by carriage, which consequently would have made travelling from home to hospital too long. Indeed, some pregnant women took more than 10 h from their home to the hospital. Pregnant women in the ethnic minority areas were usually multiparous, among whom the onset of labor would have occurred on the way to hospitals, resulting in serious health risks to both women and newborns. Despite this, the local government had no plans to initiate a bus service in these remote areas because the scattered distribution of the villagers’ houses made public transportation costly.

In Butuo county, every hospital delivery could receive TS. However, there was no TS for hospital delivery in Daofu county, so the pregnant women and their relatives had to pay for the transportation fee by themselves. As the overall cost was 100–200 Yuan, for rural poor households in Daofu county, this was a clear barrier. Nevertheless, for most pregnant women and their families, the main barrier was not the transportation cost but the lack of transport and roads.

#### 3.3.2. Costs

The delivery costs were reimbursed by several ways in Butuo county, including NRCMS, RHDS, and sometimes PFA (see Table 2). Among the latest five vaginal deliveries we investigated, all but one case received reimbursements that covered all of the hospital delivery costs (around 2000–4000 Yuan). Similarly, among the latest five cesarean deliveries, apart from one woman who did not participate in NRCMS and, thus, had to pay part of the costs by herself, the subsidies from various programs covered all of the hospital delivery cost (even if cesarean section cost more than 6000 Yuan).

The hospital delivery payment and reimbursement in Daofu were similar to that in Butuo (Table 2). NRCMS and RHDS could cover all costs of vaginal deliveries, but for cesarean section cases, the pregnant women paid 15% of the hospital fees. The amount of NRCMS reimbursement was calculated as a certain proportion of the total medical fee. After being reimbursed by NRCMS, women could also get 500 Yuan from RHDS. At times, the total refund might have exceeded the medical expenditure.

Generally, women would pay the costs up front and then get reimbursed, but the convenience of reimbursement was not guaranteed in both counties and rural women complained that it was too complicated and that they had to make several journeys to get the documents prepared.

#### 3.3.3. Religious

People in Butuo county belonged to the Yi ethnic community. Their religious beliefs of Animism had no apparent influence on women’s delivery method. However, people in Daofu county were of Tibetan nationality, who have strong religious faith in the incarnate lama. The incarnate lama was the highest religious leader in Tibetan Buddhism. Local people usually went to the temple, praying to the incarnate lama for every important decision, such as childbirth. If the divination predicted a safe home delivery, the family would keep the pregnant women at home, and refuse to send them to a hospital.

#### 3.3.4. Language

Except for a few villagers, who had received education or gone outside their region for work, and could speak Mandarin, most people in these two counties could only communicate in their own ethnic languages. Therefore, some women would worry about communication difficulties when they chose to deliver in hospitals, especially if they were not accompanied by their bilingual relatives.

### 3.4. Institution Factors for Hospital Delivery

#### 3.4.1. Quality of Hospital Conducting Midwifery Practice

In Butuo county, the county people’s hospital serves the whole county population, undertaking most of the hospital delivery work including most of the vaginal deliveries and all of the cesarean sections. There were 44 beds, six doctors, and three midwives in its obstetric department. All obstetric doctors were able to do basic obstetric operations, and 70–80 women deliver in this hospital each month. The number of deliveries in the MCH center was much less than in the people’s hospitals even they also had an obstetric department, where with four beds and four obstetric doctors basic obstetric operations (except for cesarean section) could be provided. All five township hospitals could provide obstetric services with an average of 1–2 maternity beds and 1–2 doctors in the obstetric departments.

In Daofu county, the county people’s hospital also served the whole population of this county. There were six beds and five doctors, but no midwives in the obstetric department. All obstetric doctors were able to do basic operations, and around 20 women delivered there each month. The MCH center had no pregnant patients in 2011. Despite the five beds and four obstetric doctors, it could not provide manual removal of placenta, assisted vaginal delivery, cesarean section, and neonatal respiratory support, and was not available 24 h per day and seven days per week. Only one out of four township hospitals could provide obstetric services, and there was only one maternity bed and one obstetric doctor.

#### 3.4.2. Waiting Units for Hospital Delivery

In Butuo county, there were 12 waiting units (small rooms close to hospital, pregnant women can live there until their due time) in the 12 townships, but there was no trained healthcare worker in the waiting units. Pregnant women always undertook a great amount of housework and farm work, so could not go to the waiting units for a longer time to “waste time”. There were no plans to build waiting units for hospital delivery in Daofu county.

#### 3.4.3. Maternal and Child Supplies in Hospital

Maternal and child supplies were not required to be provided by hospitals. Women needed to prepare everything, including diapers, infant formulas, thermos bottles, and infant clothes, themselves during their hospitalization in both counties. In Butuo county, HIV-positive women could get these supplies by some AIDS projects, while in Daofu county this was not available.

### 3.5. Maternal Factors

In sampled counties, the local women had traditional perceptions about delivery, the primary reasons for choosing the place of delivery were shown in Table 3. For more detailed explanations, they said “Childbirth is natural for women, there is no need to go far away for delivery, our relatives can help us, it’s very easy”. They thought childbirth could be easily assisted by experienced old women by using a pair of scissors. Their limited knowledge about pregnancy and delivery meant pregnancy complications were difficult to distinguish, and the life-threatening risks to mothers and new-born babies from non-sterilized delivery were not known.

All women were not aware of their menstrual period, neither did they have healthcare during pregnancy to estimate gestational age and calculate the expected date of birth. “When water breaks, we know it’s time for delivery, maybe just Buddha knows the time of childbirth”. By the time of labor onset, it was usually too late to go to the hospital, so women always gave birth at home.

Participants’ impression of going to hospital was equal to an operation, which was perceived as a dreadful and dangerous thing: “Doctors and nurses would give me injections and pills, which would make me very nervous. I think I am strong enough to deliver at home because I am healthy”. Women were reluctant and fearful to go to hospital because they did not want to suffer this kind of operation “If I die in a hospital, my parents and relatives would want to kill my husband because they would think that it was his fault since he sent me to a hospital”. If their husbands signed for hospital delivery and unfortunately maternal death happened, the husbands should compensate the woman’s family, but if maternal death happened at home, this would be considered as natural death and nobody would be blamed, so the husbands were also unwilling to decide on hospital delivery independently.

In both Yi and Tibetan communities, most women delivered at home because they wished to avoid the nudity involved in medical examinations or to deliver in front of strangers, so they would rather deliver at home than go to hospital. Some were concerned about their hygiene and how this would be viewed “I haven’t taken a bath for years, they would think I am dirty, it would be too embarrassing”. Only those who had experienced previous difficulties in labor were willing to go to hospital. Village health workers and some women villagers considered it as a main reason for hospital delivery.

While some people’s attitude has changed due to health education media or activities, the majority of local people, including women themselves, believed that giving birth was as ordinary as cooking or farming, so there was no need to go to hospital.

Some also said it was “ominous” (bad for children’s health and future development) if the childbirth was to be seen by strangers, “my baby would be sick with diseases such as influenza”. Others said they refused to deliver in hospital even though they could not describe exactly what the consequences were (“I cannot tell what are the exact consequences, but I think my baby will have bad luck forever”).

## 4. Discussion

As demonstrated in this study, hospital delivery can be affected by many factors related to policy, social aspects, institutional, and maternal factors, which provides a conceptual framework of the determinants of skilled birth attendance in rural ethnic communities. While we believe the findings are valuable for similar areas, especially for the same ethnic minority communities, the solutions would be applicable only in low hospital delivery rate areas of China.

Since the implementation of NRCMS and RHDS policies, the two main reimbursement sources for hospital delivery, the hospital delivery rate increased rapidly from 10.8% to 22.1% in Butuo county, and from 26.4% to 47.1% in Daofu county between 2009 and 2012. That, therefore, indicates that financial programs carried out by the government can increase hospital delivery rates. Although the medical expenses of hospital delivery could be entirely reimbursed by these two policies, problems remained, since women had to raise money for antecedent payments. This payment is a considerable amount for socioeconomically disadvantaged families and can affect their decision about hospital delivery. Furthermore, in Daofu county, TC for pregnant women and village doctors were not guaranteed and the process of reimbursement was also complicated by NRCMS and RHDS being administrated by different departments. All of these factors constitute different barriers to hospital delivery.

To address the above issues, we recommend that financial subsidies should be given to the hospitals based on their annual number of hospital deliveries and rates of reimbursement. Then hospitals will only need to verify if a pregnant woman satisfies the criteria for receiving NRCMS and RHDS. This would help to realize a “free” hospital delivery without the need of any antecedent payment. TC should also be guaranteed in every poor ethnic minority area so the families will not have to meet any costs except for medicine. Hospitals can also provide basic maternal and child supplements to increase hospital deliveries. In relation to the reimbursement procedure, NRCMS and RHDS should be integrated and managed by the health department at the county level, and women could receive subsidies in one-stop session in the delivering hospital, so hospital delivery proof would not be needed, and they can also get their reimbursements before going back home.

Lack of transport is a main barrier for hospital delivery among social factors. Ethnic minority regions are the most mountainous areas in western China with low population density, poor roads and lack of transport. To resolve this issue, many countries and regions adopted a strategy on building waiting units for hospital delivery [22,23,24], as did Butuo county. However, this strategy was not found as practical and valuable as expected. Our findings indicate the provision of funds to equip ambulances is necessary in order to provide free emergency transport for pregnant women, and investing in building good roads would also be of benefit. In addition, bilingual health workers should escort them to hospitals.

The geographic distribution of obstetric departments needs further attention. The government should implement the most cost-effective strategy by either strengthening the transport system or building hospitals in the best location, such as building township hospitals in remote areas to serve those living far from current delivery hospitals and ensuring safe delivery in remote areas by providing accessible services. Governments should also pay attention to service capacity. Most hospital deliveries occurred in the county people’s hospital because the service capacity of the county people’s hospital was significantly higher than the MCH centers and township hospitals, these two kinds of hospitals need priority support on resources and policies to improve their health service, such as more training chances for staff, more funding for organizing activities of health examination and health education to expand their influence in community.

For the potential patients, traditional lay beliefs were the main barrier to hospital delivery. Traditional beliefs were common in remote ethnic minority areas, where some said childbirth should not be seen by strangers and home deliveries were considered safe if no dystocia happened. Knowledge about reproductive health was limited. For example, many did not know when their last menstrual period took place which complicated the estimation of gestational age. Health education has been proved to contribute to improving hospital delivery in ethnic minority regions [25]. Health education activities should be held frequently to address the traditional lay beliefs regarding childbirth. Specifically, (1) health education should be included in promoting MCH strategies and programs, such as advocating hospital delivery through the promotion of free prenatal care, and providing funds for the expense of organizing health education activities; (2) health education should be organized not only by the county health department but also by the township government and local village committee; (3) the content of health education should be comprehensive including necessity and advantages of hospital delivery, basic knowledge of pregnancy, hospital delivery compensation, and the introduction of the maternal institutions; and (4) health education should be carried out by local health workers in the local language.

The focus groups were conducted with women speaking their local language and subsequently translated to the research team. It is possible that this process altered word choice if not the meaning, hence, we have not used as many or as extensive participant quotes as may be expected. In addition, the specific local situations, including health investigation, transport, religion etc., would limit the generalization of our findings. Despite these limitations, this study explores a number of potential influencing factors and the current barriers to hospital deliveries in poor ethnic minority areas, the findings can help local health governments to improve their strategies for promoting hospital delivery and, thereby, greatly improve maternal and child health. However, encouraging women to give birth in hospital must be accompanied with quality care in hospital. There are also many other factors contributing to reducing maternal deaths, and sometimes, hospital delivery might not be appropriate for rural women who live very far away from hospitals, especially that they need to head to hospitals at their due time [26,27].

## 5. Conclusions

While raising the hospital delivery rate to 80% poses a great challenge, this study reveals the influencing factors of hospital delivery in rural, poor, ethnic minority areas, including in Yi and Tibetan ethnic communities, and could thus help improve the hospital delivery rate and reduce this health inequity in China. Reimbursement procedures, transportation, hospital number and capacity, traditional conceptions are the main barriers for hospital delivery, and these factors should be the focus for practice and research. Further, local governments need to acknowledge the importance of hospital delivery, as it is critical for maternal and child health. Local governments should play an important role in solving these problems.

## Figures and Tables

**Table 1:** Characteristics of the sampled counties from questionnaires.

Indicator	Butuo County	Daofu County
Total population	172,000	55,290
Square (km^2^)	1685	7053
Average altitude (m)	2300	3500
Nationality	Yi (100%)	Tibetan (100%)
Number of townships	30	22
Number of townships whose center can be reached by vehicle	28	22
Number of townships with township hospitals	30	22
Number of township hospitals	5	4
Number of township hospitals conducting midwifery practice	5	1
Number of villages	190	158
Number of villages which can be reached by vehicle	127	154
GDP in 2011 (Yuan)	194,400	48,251
Annual per capital income in 2011 (Yuan)	3575	3303
Number of reproductive women in 2011	34,367	15,366
Number of Under-5 in 2011	18,797	2830
Number of live births in 2011	3370	539
Hospital delivery rate in 2011 (%)	22.1	47.1
Number of health facilities conducting midwifery practice	7	3
Number of women receiving facility deliveries receiving NRCMS reimbursement in 2011	698	97
Number of women receiving RHDS in 2011	698	194
Number of women receiving PFA in 2011	565	-
TS for hospital deliveries, YES or NO	YES, 100 yuan	NO

Notes: GDP: gross domestic product; NRCMS: New Rural Cooperative Medical Scheme; RHDS: Rural Hospital Delivery Subsidy; PFA: Poverty Financial Assistance; TS: Transportation subsidy.

**Table 2:** Total costs and reimbursement of the latest vaginal deliveries and cesarean deliveries.

County	Vaginal Delivery				Cesarean Delivery			
	Total Costs (¥)	RHDS (¥)	NRCMS (¥)	Out-of-Pocket (¥)	Total Costs (¥)	RHDS (¥)	NRCMS (¥)	Out-of-Pocket (¥)
Butuo	2132.70	500	991.10	640.57	11,189.27	500	7482.0	3207.27
	2447.79	500	1947.79	0.00	8296.42	500	7796.4	0.00
	3884.85	500	3384.85	0.00	11,771.77	500	11,271.8	0.00
	3822.48	500	3322.48	0.00	6795.97	500	6296.0	0.00
	3957.16	500	3457.16	0.00	9860.27	500	9360.3	0.00
Daofu	1302.24	500	802.24	0.00	3319.6	500	2135.8	683.8
	990.16	500	490.16	0.00	3785.7	500	2437.8	847.9
	907.80	500	407.80	0.00	3668.9	500	2745.9	423.0
	1009.10	500	509.10	0.00	3663.4	500	2610.4	553.0
	745.90	500	245.90	0.00	4201.5	500	3009.9	691.6

Notes: The reimbursements from PFA and TS programs were not included in the calculation because this information could not be obtained in the investigated facilities, so the individual out-of-pocket costs were the maximum estimates.

**Table 3:** Characteristics of participants in group discussions.

County	Age	Religion	Education	Parity	Delivery Place of the Latest Child	Primary Reason for Delivering at Home	Primary Reason for Delivering at Hospital
Butuo	39	Animism	Illiteracy	3	Home	“I was healthy enough”	
	41	Animism	Illiteracy	4	Home	“I was too shy”	
	24	Animism	Illiteracy	2	Home	“I don’t know Mandarin”	
	23	Animism	Illiteracy	3	Home	“It was too late”	
	42	Animism	Illiteracy	6	Home	“No one was willing to take me to hospital, there’s no transportation”	
	36	Animism	Illiteracy	3	Home	“It was already late”	
	25	Animism	Illiteracy	1	Home	“Too late to go”	
	35	Animism	Illiteracy	3	Home	“I delivered on the way to hospital, and then at home”	
	29	Animism	Illiteracy	3	Home	“I didn’t think it’s necessary”	
	37	Animism	Illiteracy	3	Home	“Too late”	
	30	Animism	Illiteracy	3	Home	“Too late”	
	25	Animism	Illiteracy	2	County hospital		“Difficult labor”
	28	Animism	Illiteracy	4	County hospital		“I thought it would be dangerous at home”
	32	Animism	Illiteracy	3	Township hospital		“Difficult labor”
	40	Animism	Illiteracy	5	County hospital		“I am HIV positive”
Daofu	39	Buddhism	Illiteracy	1	Home	“It was night”	
	28	Buddhism	Middle school	1	Home	“I was afraid to go”	
	27	Buddhism	Illiteracy	1	Home	“I delivered on the way to hospital”	
	25	Buddhism	Primary school	1	Home	“No one cared about me, and Buddha said it’s ok to be at home”	
	26	Buddhism	Illiteracy	1	Home	“Already too late”	
	19	Buddhism	Primary school	1	Township hospital		“My relative is a doctor in hospitals”
	22	Buddhism	Primary school	1	County hospital		“Anemia during pregnancy”
	22	Buddhism	Primary school	1	Township hospital		“my parents told me to”
	32	Buddhism	Primary school	1	Prefecture hospital		“I am elderly parturient”
	28	Buddhism	Illiteracy	1	County hospital		“I was worried about the baby”
	31	Buddhism	Primary school	1	County hospital		“Doctor told me to come”

## Abbreviations

The following abbreviations are used in this manuscript:

NRCMS	New Rural Cooperative Medical Scheme
RHDS	Rural Hospital Delivery Subsidy
MCH	Maternal and Child health
TS	Transportation subsidy
PFA	Poverty Financial Assistance
